# Dose dense 1 week on/1 week off temozolomide in recurrent glioma: a retrospective study

**DOI:** 10.1007/s11060-012-0832-5

**Published:** 2012-03-07

**Authors:** Walter Taal, Joyce M. W. Segers-van Rijn, Johan M. Kros, Irene van Heuvel, Carin C. D. van der Rijt, Jacoline E. Bromberg, Peter A. E. Sillevis Smitt, Martin J. van den Bent

**Affiliations:** 1 Department Neurology/Neuro-oncology Unit, Erasmus MC University Hospital/Daniel den Hoed Cancer Center, Groene Hilledijk 301, 3075 EA Rotterdam, The Netherlands; 2 Present Address: Department of Neurology, Haga Hospital, Leyweg 275, 2545 CH The Hague, The Netherlands; 3 Deparment of Pathology, Erasmus MC University Hospital, ′s-Gravendijkwal 230, 3015 CE Rotterdam, The Netherlands; 4 Deparment of Medical Oncology, Erasmus MC University Hospital/Daniel den Hoed Cancer Center, Groene Hilledijk 301, 3075 EA Rotterdam, The Netherlands

**Keywords:** Glioma, Glioblastoma, Dose dense temozolomide, 1p, 19q, Chemotherapy, Brain tumors, Astrocytoma, Oligodendroglioma

## Abstract

Alternative temozolomide regimens have been proposed to overcome O^6^-methylguanine-DNA methyltransferase mediated resistance. We investigated the efficacy and tolerability of 1 week on/1 week off temozolomide (ddTMZ) regimen in a cohort of patients treated with ddTMZ between 2005 and 2011 for the progression of a glioblastoma during or after chemo-radiation with temozolomide or a recurrence of another type of glioma after radiotherapy and at least one line of chemotherapy. Patients received ddTMZ at 100–150 mg/m^2^/d (days 1–7 and 15–21 in cycles of 28-days). All patients had a contrast enhancing lesion on MRI and the response was assessed by MRI using the RANO criteria; complete and partial responses were considered objective responses. Fifty-three patients were included. The median number of cycles of ddTMZ was 4 (range 1–12). Eight patients discontinued chemotherapy because of toxicity. Two of 24 patients with a progressive glioblastoma had an objective response; progression free survival at 6 months (PFS-6) in glioblastoma was 29%. Three of the 16 patients with a recurrent WHO grade 2 or 3 astrocytoma or oligodendroglioma or oligo-astrocytoma without combined 1p and 19q loss had an objective response and PFS-6 in these patients was 38%. Four out of the 12 evaluable patients with a recurrent WHO grade 2 or 3 oligodendroglioma or oligo-astrocytoma with combined 1p and 19q loss had an objective response; PFS-6 in these patients was 62%. This study indicates that ddTMZ is safe and effective in recurrent glioma, despite previous temozolomide and/or nitrosourea chemotherapy. Our data do not suggest superior efficacy of this schedule as compared to the standard day 1–5 every 4 weeks schedule.

## Introduction

Gliomas are the most common primary brain tumors in adults and are usually classified and graded according to the World Health Organisation (WHO). Currently, chemotherapy is standard of care for all diffuse gliomas, either at first diagnosis or at first recurrence [1–5]. The most frequently used agents are temozolomide (TMZ) and nitrosoureas. Treatment options for patients failing radiotherapy and a first line of alkylating or methylating chemotherapy are limited. The cytotoxic effect of TMZ is mediated primarily via methylation at the O^6^ position of guanine. One of the main mechanisms of tumor resistance to TMZ is thought to be mediated by O^6^-methylguanine-DNA methyltransferase (MGMT) [6]. Evidence supporting this role of MGMT comes from clinical studies indicating that hypermethylation of the promoter of MGMT is associated with improved tumor response and survival in patients with GBM [7, 8]. Because of the more continuous exposition with ddTMZ it has been assumed that dose dense temozolomide (ddTMZ) schedules could overcome MGMT dependent resistance against TMZ by a more effective depletion of deplete intracellular levels of the DNA repair enzyme, MGMT [9]. Studies using ddTMZ show it is well tolerated and generally safe, also when given in higher monthly doses and in patients who have previously received TMZ [10–18]. We used the 1 week on/1 week off TMZ regimen (ddTMZ) for patients with relapsing GBM or other glioma after prior TMZ or nitrosourea chemotherapy to study the efficacy and toxicity of ddTMZ in heavily pre-treated patients with high-grade glioma.

## Materials and methods

Data of all diffuse glioma patients treated with ddTMZ after prior chemotherapy in our center were retrospectively collected. The study was approved by the local institutional review board. Patients were included in this study if they had a histologically confirmed low-grade glioma or high-grade glioma, with a progressive and measurable enhancing tumor on the MRI (diameter >2 cm), relapsing after prior radiotherapy and at least one line of chemotherapy, and had concluded RT at least 3 months prior to the diagnosis of progression. We collected data about patient characteristics, tumor characteristics, prior treatment, number of ddTMZ cycles, use of dexamethason, adverse effects, reason of discontinuation, and further treatments. According to histology three categories of patients were distinguished: patients treated with ddTMZ for a progressive primary GBM after radiotherapy and TMZ (group A); patients with recurrent astrocytoma WHO grade 2 or 3 (group B), or recurrent oligo-astrocytoma WHO grade 2 or 3, without 1p and 19q loss; and patients with progressive WHO grade 2 or 3 oligodendroglioma or oligo-astrocytoma with 1p and 19q loss (group C). WHO grade 2 tumors were combined with WHO grade 3 tumors because all patients had contrast enhancing lesions on the MRI scan at the time of treatment with ddTMZ, suggesting malignant dedifferentiation of the WHO grade 2 tumors. Furthermore, a previous study with TMZ in recurrent WHO grade 2 astrocytoma, with enhancement on the MRI-scan, at our institution has shown similar results to the pilot trial of TMZ in recurrent WHO grade 3 gliomas (PFS at 12 months 25% vs. 24%). [3, 4].

Patients received TMZ on day 1–7 and on day 15–21 of a 28-day cycle for up to 12 cycles or until documented disease progression or unacceptable toxicity. The starting dose of the TMZ was 100 mg/m^2^/day. In the absence of toxicity or only CTCAE grade 1 toxicity during the first two treatment weeks the dose was escalated, in two steps to dose level 1 (150 mg/m^2^/day; Table 1 for dose levels). Toxicity was evaluated according to the Common Terminology Criteria for Adverse Events (CTCAE; version 3.0). In case of hematological toxicity grade 4 or non-hematological toxicity grade 3, the dosage TMZ of the next cycle was reduced with 1 dose level. In case of CTCAE grade 4 non-hematological toxicity, the patient stopped treatment. In case of a grade 4 hematological toxicity or a grade 3 non-hematological toxicity at dose level 4 (75 mg/m^2^/day), the patient went off treatment. In case of dose reductions, dose re-escalation was not allowed. Blood examinations were done on day 15 and day 29 and when platelets were above 100*10^9^/l and neutrophils counts above 1.5*10^9^/l, the following 7 days TMZ was administered. Otherwise treatment was postponed until recovery to ≤CTCAE grade 1 and/or platelets were above 100*10^9^/l. The treatment was stopped if it had to be postponed for more than 2 weeks.

**Table 1:** Dose levels of dose dense temozolomide

Dose level	Daily temozolomide dose (mg/m^2^/day)	Dose temozolomide per cycle (mg/m^2^)
1	150	2100
2	125	1750
3	100	1400
4	75	1050

The objectives of the study were the assessment of progression free survival at 6 (PFS-6) and 12 (PFS-12) months, objective response rate (ORR), overall survival (OS), and toxicity. OS was calculated from the start of the TMZ treatment to the date of death. PFS was calculated from the start of the TMZ until the date of progression or death. Response was assessed using RANO criteria [19, 20]. Both complete and partial responses were considered objective responses. Clinical evaluation was done every 4 weeks and MRI was made every 12 weeks or in case of neurological deterioration. Response to treatment was reviewed as part of this analysis (W.T.). In this exploratory analysis, no adjustments were made for multiple testing.

## Results

Between June 2005 and June 2011, 53 patients were treated with ddTMZ for the progression of a glioma in our center. Twenty-four patients were treated for a recurrent GBM (group A), 16 patients were treated for a recurrence of a WHO grade 2 or 3 astrocytoma, oligodendroglioma, or oligo-astrocytoma, without combined 1p and 19q loss, with a contrast enhancing lesion on MRI (group B) and 13 patients were treated for a recurrence of a WHO grade 2 or 3 oligodendroglioma or oligo-astrocytoma with combined 1p and 19q loss, with a contrast enhancing lesion on MRI (group C). Tables 2 and 3 show the patient characteristics of the 53 patients. All GBM patients progressed after chemo-irradiation with TMZ, except for one patient who had progression after radiotherapy and during the 6th cycle of 1st line standard day 1–5 every 4 weeks schedule TMZ chemotherapy. Five GBM patients treated with chemo-irradiation progressed directly after six cycles of adjuvant TMZ; all other patients had a TMZ free interval, before the start of ddTMZ. Six patients with a recurrent primary-GBM received a second line of therapy after chemo-irradiation: dendritic-cell therapy (1), cediranib (1), lomustine combined with cediranib (2), and, imatinib combined with hydroxyurea (2).

**Table 2:** Characteristics of patients treated with dose dense temozolomide for a progressive glioma after radiotherapy and 1 or 2 lines of chemotherapy

Characteristic	All patients No. (%) of patients, *n* = 53	Group A No. (%) of patients, *n* = 24	Group B No. (%) of patients, *n* = 16	Group C No. (%) of patients, *n* = 13
Age, years				
Median	49	52	43	44
Range	31–74	31–74	32–61	33–60
Sex				
Male	38 (72%)	14 (58%)	13 (81%)	11(85%)
Female	15 (28%)	10 (42%)	3 (19%)	2 (15%)
First symptom				
Epilepsy	36 (68%)	11 (46%)	13 (81%)	12 (92%)
Other	17 (32%)	13 (54%)	3 (19%)	1 (8%)
WHO-PS				
0	15 (28%)	6 (25%)	5 (31%)	4 (31%)
1	27 (51%)	16 (67%)	7 (44%)	4 (31%)
2	11 (21%)	2 (8%)	4 (25%)	5 (38%)
WHO histology grade at first operation				
2			10 (63%)	6 (46%)
3			6 (37%)	7 (54%)

*WHO* World Health Organisation, *PS* Performance Score

*Group A* patients with recurrent primary glioblastoma, *Group B* patients with recurrent WHO grade 2 or 3 astrocytoma or oligodendroglioma or oligoastrocytoma without combined 1p and 19q loss and with a contrast enhancing lesion on MRI, *Group C* patients with recurrent WHO grade 2 or 3 oligodendroglioma or oligoastrocytoma with combined 1p and 19q loss and with a contrast enhancing lesion on MRI

**Table 3:** Previous treatments of patients treated with dose dense temozolomide for a progressive glioma after radiotherapy and 1 or 2 lines of chemotherapy

Characteristic	All patients No. (%) of patients, *n* = 53	Group A No. (%) of patients, *n* = 24	Group B No. (%) of patients, *n* = 16	Group C No. (%) of patients, *n* = 13
ddTMZ as 2nd line of chemotherapy	40 (75%)	19 (79%)	11 (69%)	10 (77%)
ddTMZ as 3nd line of chemotherapy	13 (25%)	5 (21%)	5 (31%)	3 (23%)
Second operation	15 (28%)	3 (13%)	6 (38%)	6 (46%)
Third operation	1 (2%)	0	0	1 (8%)
Time between start last RT and start ddTMZ, median (range) in months	24 (5–197)	19 (8–75)	18 (5–197)	45 (10–93)
Time between last chemotherapy and start ddTMZ, median (range) in months	10 (0–94)	5 (0–67)	13 (1–92)	17 (1–94)
Prior 1st line treatment	53 (100%)	24 (100%)	16 (100%)	13 (100%)
RT/TMZ	27 (51%)	23 (96%)	3 (19%)	1 (8%)
TMZ	6 (11%)	1 (4%)	5 (31%)	0
PCV	20 (38%)	0	8 (50%)	12 (92%)
Prior 2nd line treatment	13 (25%)	5 (21%)	5 (31%)	3 (23%)
RT/TMZ	1 (2%)	0	1 (6%)	0
TMZ	5 (9%)	0	3 (19%)	2 (15%)
PCV	2 (4%)	0	1 (6%)	1 (8%)
Other	5 (9%)	5 (21%)	0	0

*ddTMZ* dose dense temozolomide, *RT* radiotherapy

*Group A* patients with recurrent primary glioblastoma, *Group B* patients with recurrent WHO grade 2 or 3 astrocytoma or oligodendroglioma or oligoastrocytoma without combined 1p and 19q loss and with a contrast enhancing lesion on MRI, *Group C* patients with recurrent WHO grade 2 or 3 oligodendroglioma or oligoastrocytoma with combined 1p and 19q loss and with a contrast enhancing lesion on MRI

The median number of cycles of ddTMZ was 4 (range 1–12), three patients completed 12 cycles. Most patients stopped because of tumor progression. One patient stopped because of unrelated cholecystitis and elevated transaminases. Eight patients discontinued ddTMZ because of toxicity: grade 4 thrombocytopenia (1), persistent grade 2 or grade 3 fatigue (5), grade 3 elevated transaminases (1), and grade 3 allergic skin reaction (1). Five patients who stopped because of fatigue continued TMZ in regular regimen of day 1–5 in a 28 day cycle and tolerated this well. In 25 patients, CD4+ lymphocytes counts were monitored; 14 (56%) of these patients developed a grade 3 CD4+ lymphopenia (<0.2*10^9^/l) and 6 (24%) of these patients developed a grade 4 CD4+ lymphopenia (<0.05*10^9^/l). All patients with grade 3/4 CD4+ lymphopenia received prophylactic cotrimoxazol. None of these patients developed a pneumocystis carinii pneumonia. Two of the patients with a grade 4 CD4+ lymphopenia developed a *pneumocystis carinii* pneumonia, prior to routine monitoring of CD4+ counts, from which they fully recovered.

Fifty-two patients were evaluable for response. In the patient with cholecystitis, no follow-up imaging was done. The PFS-6, the ORR (complete and partial response) and median OS for the three groups of patients are shown in Table 4. The median interval (and range) between the prior chemotherapy and the start of the ddTMZ was 5 months (0–67 months) in group A, 12.5 months (range 1–92) in group B and 17 months (range 1–94) in group C. The patients without 1p and 19q loss (group A and B) that started with the ddTMZ within 3 months of the previous chemotherapy (12 out of 40 patients) had a lower PFS-6 compared to the patients with a chemotherapy free interval of more than 3 months (PFS-6 8% vs. 43%; Fisher exact test 0.033).

**Table 4:** Outcome of patients treated with dose dense temozolomide for a progressive glioma after radiotherapy and 1 or 2 lines of chemotherapy

Outcome	All patients *n* = 53	Group A *n* = 24	Group B *n* = 16	Group C *n* = 13
PFS-6	40%	29%	38%	62%
PFS-12	13%	13%	13%	15%
Median OS	9 months	6 months	9 months	19 months
CR + PR	17%	8%	19%	33% (4:12)

*PFS-6* progression free survival at 6 months, *PFS-12* progression free survival at 12 months, *OS* overall survival, *CR* complete response, *PR* partial response

*Group A* patients with recurrent primary glioblastoma, *Group B* patients with recurrent WHO grade 2 or 3 astrocytoma or oligodendroglioma or oligo-astrocytoma without combined 1p and 19q loss and with a contrast enhancing lesion on MRI, *Group C* patients with recurrent WHO grade 2 or 3 oligodendroglioma or oligo-astrocytoma with combined 1p and 19q loss and with a contrast enhancing lesion on MRI

## Discussion

In this group of 53 chemotherapy and radiotherapy pre-treated gliomas, ddTMZ showed activity. Although this is a retrospective study with a limited sample size, our results are also comparable to other studies on dose-dense TMZ in recurrent gliomas [12–17].

However, in all our groups the observed activity is well within the range of previous reports on standard dosing TMZ. The PFS-6 of 29% (95%-CI 11–47%) (Table 4; group A) in GBM is within the range of the pivotal standard dose phase II TMZ trials in recurrent GBM. (PFS-6: 19–24%) [21–23] More in particular, Brandes et al. described a 24% (95% CI 14–42%) for 2nd line standard dosing TMZ in recurrent GBM. The results in group B (recurrent WHO grade 2 or 3 astrocytoma or oligodendroglioma without 1p and 19q loss) are comparable to the 2nd line results in the pivotal phase 2 trial in recurrent anaplastic astrocytoma or anaplastic oligo-astrocytoma (PFS-6 44% versus PFS-6 38% in the present series, Table 4) [4].

The PFS-6 in group B and C (All recurrent non-primary GBM’s, with a contrast enhancing lesion on MRI at the start of the ddTMZ) is higher than the PFS-6 found in the EORTC study 26972 in recurrent oligodendroglioma, with or without combined 1p/19q loss after first line chemotherapy (Table 4: PFS-6 38–62% vs. 29%), although the PFS-12 is comparable (Table 4: PFS-12 13–15% vs. 11%) [24]. Data on second line TMZ in recurrent oligodendroglial tumors with combined 1p/19q loss are scarce, Kouwenhoven et al. [25] reported only one responder in nine patients treated after prior procarbazine, lomustine, and vincristine chemotherapy, but PFS-6 was not reported.

Almost none of the patients with a primary GBM or WHO grade 2 or 3 glioma without combined 1p/19q loss (group A and B) with a chemotherapy free interval of 3 months or less before the start of the ddTMZ had a good outcome (PFS-6 8% vs. 43%; Fisher exact test 0.033). Similar to the results of Perry et al. [10] on metronomic TMZ, ddTMZ is not effective in patients with progressive disease within 3 months of previous chemotherapy. The patients with a dedifferentiated glioma with combined 1p/19q loss were left out of this analysis, because these patients have a completely different prognosis and response to chemotherapy and only two patients in this group had progressive disease within the 3 months before the start of the ddTMZ. The single patient relapsing during standard TMZ and responding to ddTMZ had a progression free survival of 48 months up till now, suggesting he didn’t have real tumor progression at the time of ddTMZ. Probably the enhancement on MRI in this patient was caused by radionecrosis 45 months after RT. Since all patients started ddTMZ relatively long after (chemo-)irradiation (median time between start last RT and start ddTMZ 24 months, range 5–197; Table 3), it is unlikely that pseudoprogression played a role in this study [26].

Dose dense TMZ appears more toxic than the standard dosing regimen of TMZ. Five patients were switched to the standard day 1–5 every 4 weeks TMZ because of fatigue. After switch their fatigue improved. Two patients developed PCP infections before routine monitoring of CD4+ counts, none of the monitored patients (who received PCP prophylaxis with cotrimoxazol in case CD4+ counts decreased below 0.2*10^9^/l) developed a PCP infection. Data from available phase 2 trials investigating ddTMZ in gliomas indicate a high incidence of lymphopenia, especially in patient treated with the 3 weeks on/1 week off regimen [12, 14, 16, 17, 27]. In melanoma patients treated with daily TMZ for 6 weeks out of every 8-week cycle, a high incidence of lymphopenia and an increased risk of opportunistic infections were reported [28]. Clearly, patients who receive ddTMZ are at risk to develop *Pneumocystis carinii* pneumonia, and prophylaxis is indicated in patients who develop lymphopenia or low CD4+ counts.

Although this study has a limited number of patients and is retrospective, it however seems from these results that ddTMZ is an effective treatment for patients with a recurrence of GBM or otherwise heavily pre-treated gliomas, albeit with an increase in toxicity. Whether it is more effective than the standard 5 of 28-day regimen remains unclear.

Although administration of ddTMZ regimens causes more pronounced depletion of MGMT in peripheral blood mononuclear cells [9], the effects of ddTMZ on MGMT activity in brain tumor tissue and its impact on clinical outcome remain unclear. A study from the United Kingdom, comparing standard day 1–5 every 4 weeks TMZ with a ddTMZ schedule, (given in a 3 weeks on/1 week off schedule) failed to show any benefit of ddTMZ in high-grade glioma recurrent after RT only in comparison to the standard day 1–5 every 4 weeks schedule [29]. Of note, although these patients were chemotherapy naïve, one may assume that two-thirds of patients would have an unmethylated MGMT promoter. Thus, if ddTMZ would have been effective in overcoming that resistance, one would expect at least some trend toward a more favorable outcome in ddTMZ treated patients. The recently reported RTOG 0525 trial on newly diagnosed GBM also failed to produce superior outcome of ddTMZ in newly diagnosed GBM (and regardless of the MGMT promoter status) [30]. This casts further doubt on the usefulness of intensified dosing regimen. Future trials into ddTMZ regimens require a control arm with a standard dosing regimen.
